# Supplementary material for: Efficacy of Nasal High-Flow Oxygen Therapy in Chronic Obstructive Pulmonary Disease Patients in Long-Term Oxygen and Nocturnal Non-Invasive Ventilation during Exercise Training
Source: Healthcare (Basel). 2022 Oct 11;10(10):2001. doi: 10.3390/healthcare10102001 (PMC9601581; doi:10.3390/healthcare10102001)
Supplement: Supplementary file 1 [file healthcare-10-02001-s001.zip › healthcare-1915116-supplementary.pdf]

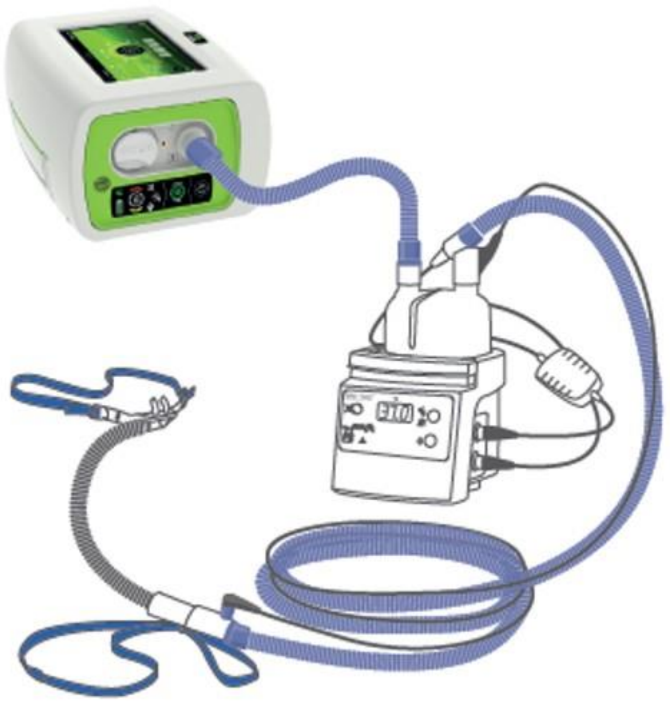

Figure S1. EOVE-150 device set for therapy with high flows.

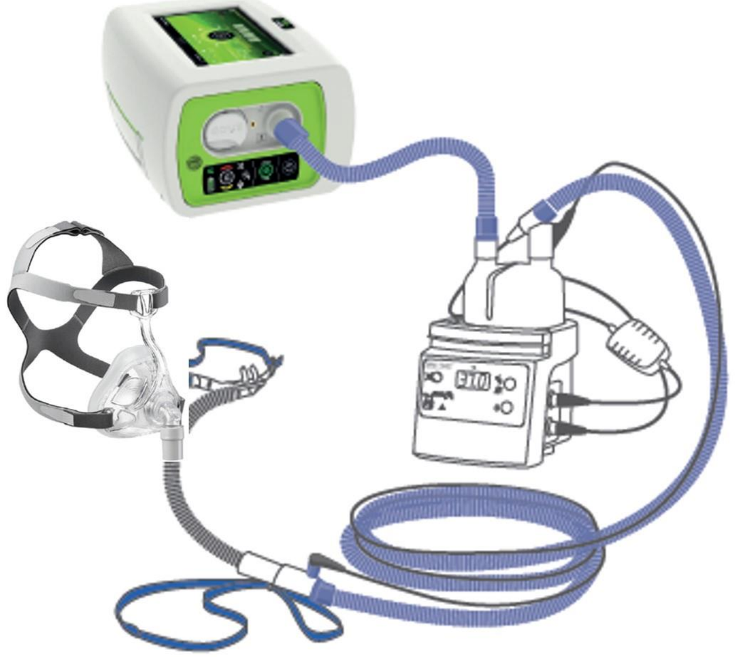

Figure S2. EOVE-150 device set for therapy with Non-Invasive Ventilation.

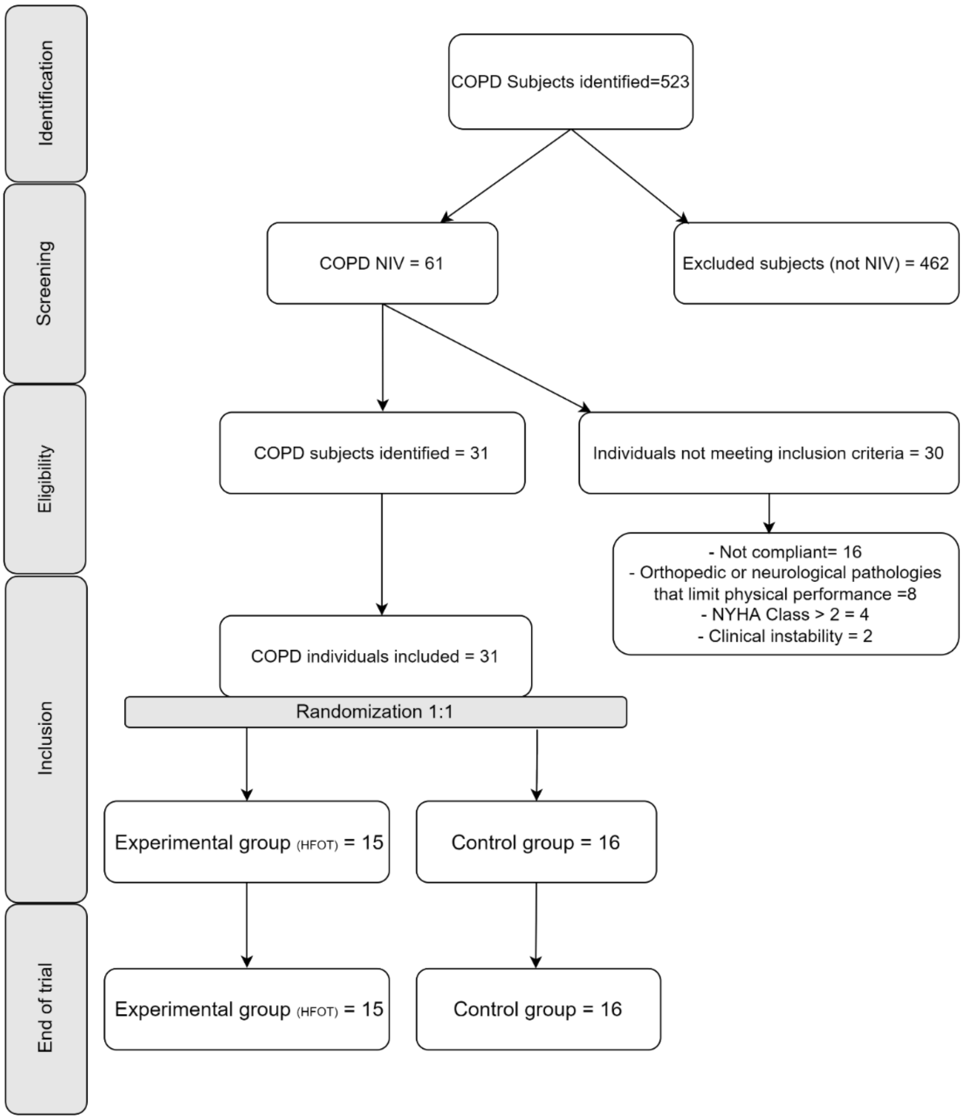

Figure S3. Flow Chart.
